# Acute Lung, Heart, Liver, and Pancreatic Involvements with Hyponatremia and Retinochoroiditis in a 33-Year-Old French Guianan Patient

**DOI:** 10.1371/journal.pntd.0001802

**Published:** 2012-10-25

**Authors:** Matthieu Groh, Alexandra Faussart, Isabelle Villena, Daniel Ajzenberg, Bernard Carme, Magalie Demar, Véronique Joly, Sandrine Houze, Stéphane Simon, Dominique Aubert, Cécile Charlois-Ou, Patrick Yeni

**Affiliations:** 1 Department of Infectious Diseases, Hopital Bichat, Paris Diderot University, Paris, France; 2 Department of Parasitology and Mycology, Hopital Bichat, Paris Diderot University, Paris, France; 3 National Reference Centre on Toxoplasmosis and Biological Resource Centre Toxoplasma, EA3800, Department of Parasitology and Mycology, CHU Maison Blanche, University of Reims Champagne-Ardenne, Reims, France; 4 INSERM UMR 1094, Tropical Neuroepidemiology, Department of Parasitology and Mycology, University of Limoges, Limoges, France; 5 EA 3593, Department of Parasitology and Mycology, Cayenne Hospital, University of the Antilles and Guyana, Cayenne, French Guiana; Emory University, United States of America

## Description of Case

A 33-year-old man living in Cayenne, French Guiana, was admitted in the Bichat hospital in Paris with a 3-week history of fever associated with dyspnea and confusion. A chest X-ray previously made in French Guiana showed an alveolar infiltrate of the middle lobe. Amoxicillin and then doxycycline were administered, but did not alleviate the symptoms. Physical examination revealed a loss of 4 kilograms in the last month, confusion, hypotension (98/63 mmHg), tachycardia (126 beats per minute), a red but painless left eye, congestive heart failure, fine crackles in the right lung field, and a one-centimetre-wide left axillary node.

Haematological blood tests revealed a haemoglobin level of 137 g/l with signs of haemolysis (elevated lactic dehydrogenase, low haptoglobin), a total white blood cell count of 11.1×10^9^/L (69% granulocytes, 21% lymphocytes), and thrombocytosis (392×10^9^/L platelets). The blood film showed stigmata of mononucleosis syndrome. Blood chemistry revealed hyponatremia, mild hepatitis, pancreatitis, and elevated cardiac markers. Table 1 summarizes biological data.

**Table 1:** Laboratory findings.

Biological Characteristics	Day 1	Day 6	Day 19	Day 41	Day 60	Normal Values
Natremia (mmol/L)	127	125	133	140	140	135–145
Blood osmolarity (mosmol/L)	259	na^1^	na	na	na	300–310
Urine osmolarity (mosmol/l)	508	na	na	na	na	500–100
Thyroid stimulating hormone (mIU/L)	1.9	na	na	na	na	0.3–5.0
Cortisol^2^ (nmol/L)	598	na	na	na	na	138–745
Aspartate aminotransferase (IU/L)	118	83	233	55	46	<40
Alanine aminotransferase (IU/L)	97	86	189	46	45	<40
Gamma-glutamyl transpeptidase (IU/L)	127	76	245	27	27	<58
Alkaline phosphatase (IU/L)	66	51	119	34	na	<34
Total bilirubin (micromol/L)	17	13	9	10	na	<17
Troponin (microg/L)	1.59	0.36	na	0.05	na	<0,6
Brain natriuretic peptide (ng/L)	1,510	274	na	258	na	<100
Lipase (IU/L)	503	1,142	771	283	0	<60
Lactic dehydrogenase (U/L)	1,978	na	na	na	na	<470
Haptoglobin (g/L)	0.1	na	na	na	na	0.64–1.7
Creatinine phosphokinase (IU/L)	340	na	na	na	na	10–200
Anti-*Toxoplasma* IgG (IU/mL)^3^	68	147	199	785	294	positive if >4 IU/mL
Anti-*Toxoplasma* IgM (index)^3^	42.7	45	38	45	45	positive if >0.75
Anti-*Toxoplasma* IgG Avidity index^4^	0.04	na	na	na	na	recent infection if <0.3
*Toxoplasma* PCR in blood	positive	na	na	na	negative	negative

1 Not available.

2 8:00 am measurement.

3 Vidia Biomérieux.

4 Vidas Biomérieux.

Electrocardiogram showed sinus tachycardia. Transthoracic echocardiography revealed an altered left ventricular ejection fraction (35% estimation with Simpson's method), global hypokinesia, but no signs of endocarditis. Abdominal computed tomography (CT) scanner was normal.

## What Further Investigations Would You Perform to Make Etiological Diagnosis?

There are many diagnoses that can lead to subacute fever, hepatitis, pancreatitis, and pneumonia in a patient coming from the Amazonian region. Blood cultures, thick and thin smears for malaria, and intradermal purified protein derivative test for tuberculosis were negative. *Mycobacterium tuberculosis* was not detected on gastric aspirates. Urines were sterile, and antigen detection for *Streptococcus pneumoniae* and *Legionella pneumophila* was negative. *HIV*, *hepatitis viruses* (*A*, *B*, and *C*), *Coxiella burnetti*, *Rickettsia conorii*, *Salmonella typhi and paratyphi*, *Brucella sp.*, *Dengue fever*, and *Mumps virus* serologies were also negative. *Chlamydia pneumoniae* and *Mycoplasma pneumonia* serologies were positive with the presence of both IgM and IgG. *Epstein-Barr virus (EBV)*-IgM and IgG anti-VCA antibodies were also found, but detection of *EBV* and *Cytomegalovirus* (*CMV*) DNA by polymerase chain reaction (PCR) was negative. Since the patient had severe cardiac dysfunction, Chagas' disease was also looked for, but antibodies against *Trypanosoma cruzi* were absent. Cerebrospinal fluid analysis revealed 8 cells/mm3, hypoglycorrhacchia (2.3 mmol/L with venous glycaemia 5.5 mmol/L), hyperproteinorrachia (0.54 g/L), sterile culture, and negative pneumococcal and cryptococcal antigen detection. *Toxoplasma gondii*, *Herpes simplex viruses* (*type-1* and *-2*), *EBV*, and *Varicella-zoster virus* DNA were not detected by PCR in the cerebrospinal fluid.

## What Finally Led to Diagnosis?

The patient reported the consumption of semi-raw game (Brazilian Tapir, *Tapirus terrestris*, locally known as Maïpouri) 3 weeks before the beginning of symptoms, pieces of which were still kept frozen.

Ophthalmologic examination showed a cornea ulcer associated with multiple foci of retinochoroiditis in the left eye, which is suggestive of toxoplasmosis.

Systematic exploration of the causes of mononucleosis syndrome led (besides *HIV*, *EBV*, and *CMV* serologies) to *T. gondii* serology, which was positive with the presence of IgG (63 IU/ml), high levels of IgM (index of 42.7, Vidia Biomerieux), and IgA (12/12 with ISAGA Biomerieux test). The avidity of anti-toxoplasmic IgG was low (4% Vidas Biomerieux). These data suggested recent toxoplasmosis infection. Table 1 summarizes serological data.

## How Can Hyponatremia and Hemolysis Be Explained?

Since blood osmolarity was low (259 mosmol/L) and urine osmolarity was high (508 mosmol/L), the syndrome of inappropriate antidiuretic hormone secretion (SIADH) was suspected. Cortisol response to synacthen test and level of serum thyroid-stimulating hormone were normal. No medication causing hyponatremia as a side effect was taken. Brain magnetic resonance imaging was normal, and full-body CT scan did not suspect cancer. SIADH diagnosis was confirmed and related to pneumonia.

There were no schizocytes on the blood film. Direct Coombs' test and hemoglobin electrophoresis were negative or normal. Pyruvate kinase deficiency and Minkowski-Chauffard disease were also looked for but excluded. Glutathione stability test revealed mild g6pd deficiency (7% g6pd activity). The patient, though living in French Guiana, was of Chinese descent, and g6pd deficiency is frequent in Asia. Amoxicillin and doxycycline are suitable for g6pd patients, and we can imagine that sepsis favored haemolysis.

## How Would You Manage the Patient?

When *C. pneumonia* and *M. pneumoniae* serologies came back positive, macrolide therapy (roxithromycine 300 mg/day) was started. However, physicians doubted that these serological data could explain the whole symptoms. Three weeks after the first serologies, *C. pneumonia* and *M. pneumoniae* antibodies' rates did not rise, pointing out a cross-serological reactivity with *T. gondii* antibodies.

Since the patient was in a critical condition and despite mild g6pd deficiency, gold standard anti-toxoplasmosis treatment [1] was promptly started after the serological diagnosis of recent *T. gondii* infection. The patient's condition improved with pyrimethamine (100 mg/day for the first 2 days followed by 50 mg/day), sulfadiazine (6 g/day), and folinic acid (25 mg/day). There was no increased haemolysis with sulfadiazine, but this drug was stopped and replaced by clindamycin (2.4 g/day) due to hepatotoxicity. Anti-*Toxoplasma* drugs were given for a total of 6 weeks.

A salt-free diet, furosemide, and increased doses of perindopril and bisoprolol were also administered. Heart rate diminished, congestive heart failure symptoms disappeared, and brain natriuretic peptide and troponin levels normalized. Periodic transthoracic echocardiogram evaluations were performed and LVEF measures reached 45% at the patient's discharge.

When furosemide was stopped, and thanks to fluid deprivation, natremia normalized. The patient recovered his full intellectual abilities.

With anti-*Toxoplasma* treatment and Vitamin A ointment, cornea ulcer and chorioretinitis healed without visual aftereffect.

Lipase rose to 17N but decreased under antiparasitic therapy.

## Additional Molecular and Parasitological Investigations

A quantitative PCR-based assay detected the 200- to 300-fold repetitive 529 bp DNA fragment of *T. gondii* in a blood sample collected 3 days after admission. This blood sample was also inoculated into mice. Four weeks post-inoculation, all mice tested positive for *T. gondii* antibodies and showed signs of disease due to a virulent *T. gondii* strain.

All surviving inoculated mice were sacrificed and numerous *T. gondii* cysts were microscopically observed in the brain samples. Cell cultures were inoculated with trypsinized cysts of *T. gondii*, and the strain was named BRC TgH41001.

Genotyping analysis with 15-microsatellite markers [2] of the BRC TgH41001 strain was performed on DNA extracted from cysts collected at first isolation in mice. In each pair, one primer was 5′-end labelled with fluorescein (6-FAM, HEX, or NED) to allow sizing of PCR products with an automatic sequencer. PCR was carried out in a 25-µL-reaction mixture consisting of 12.5 µL of 2X QIAGEN Multiplex PCR Master Mix (Qiagen) and 5 pmol each primer. Cycling conditions were 15 min at 95°C, 30 s at 94°C, 3 min at 61°C, 30 s at 72°C (35 cycles), and 30 min at 60°C. PCR products were diluted in desionised formamid with a dye-labeled size standard (ROX 500, Applied Biosystems) and electrophoresed using an automatic sequencer (Abiprism 3100, Applied Biosystems). The sizes of the alleles in base pairs (bp) were estimated using GeneMapper analysis software (version 4.0, Applied Biosystems).

Total DNA was extracted on eight samples of the frozen Maïpouri meat and submitted to a quantitative PCR-based assay targeting the 200- to 300-fold repetitive 529 bp DNA fragment of *T. gondii*, but only one tested repeatedly positive for *T. gondii* DNA at low concentration (cycle thresholds of 35.74 and 36.05). The genotyping analysis with 15 single-copy microsatellite markers was attempted on this positive DNA sample but was unsuccessful because the amount of *T. gondii* DNA was too low for a successful amplification of the genetic markers.

The genotyping data of the BRC TgH41001 strain and 12 reference strains are reported in Table 2. Phylogenetic analyses of *T. gondii* strains have shown that those from the French Guianan rainforest are of special significance because they are genetically highly divergent from the ones found elsewhere in the world [3] and even from those circulating along the coastline of French Guiana [4]. The genotyping analysis of TgH41001 strain supports this viewpoint. This strain possesses a specific combination of microsatellite alleles (such as alleles 203 and 246 at *TgM-A* and *W35* markers, respectively), which to date has been described only in the atypical strains from the Amazonian rain forest (Daniel Ajzenberg, personal communication).

**Table 2:** Genotyping results of BRC TgH41001 strain and 12 reference strains with 15 microsatellite markers.

				Microsatellite Markers														
Type	Isolate^1^	Origin	Host	*TUB2*	*W35*	*TgM-A*	*B18*	*B17*	*M33*	*IV.1*	*XI.1*	*M48*	*M102*	*N60*	*N82*	*AA*	*N61*	*N83*
Atypical	BRC TgH41001	French Guiana	Human	291	246	203	158	346	169	272	356	229	172	142	105	261	105	316
I	CT1	USA	Cow	291	248	209	160	342	169	274	358	209	168	145	119	265	87	306
II	PTG	USA	Sheep	289	242	207	158	336	169	274	356	215	174	142	111	265	91	310
III	CTG	USA	Cat	289	242	205	160	336	165	278	356	215	190	147	111	269	89	312
Atypical	COUGAR	Canada	Cougar	289	242	205	158	336	169	274	354	219	174	151	119	259	79	332
Atypical	TgCatBr1	Brazil	Cat	289	242	205	160	342	165	278	358	233	164	147	111	316	89	308
Atypical	TgCatBr3	Brazil	Cat	289	242	205	160	348	165	278	356	213	190	142	111	263	113	312
Atypical	TgCatBr5	Brazil	Cat	291	242	205	160	362	165	278	356	237	174	140	111	265	89	314
Atypical	RUB	French Guiana	Human	289	242	205	170	360	167	274	356	223	190	142	109	259	85	312
Atypical	VAND	French Guiana	Human	291	242	203	162	344	167	276	356	217	170	142	113	277	91	308
Atypical	BRC TgH18001	French Guiana	Human	289	246	203	160	344	167	272	356	229	176	142	113	263	85	312
Atypical	BRC TgH18002	French Guiana	Human	289	246	203	160	337	165	274	356	209	172	136	111	251	109	310
Atypical	BRC TgH18003	French Guiana	Human	291	242	203	160	339	165	272	358	221	174	138	107	277	95	312

1 PTG is a clone of the ME49 strain; CTG is also known as CEP or C strain; COUGAR is also known as TgCgCa1 or COUG strain; BRC TgH18001 is also known as GUY-DOS or GUY-2001-DOS strain; BRC TgH18002 is also known as GUY-KOE or GUY-2002-KOE strain; BRC TgH18003 is also known as GUY-MAT or GUY-2002-MAT.

## Why Was the Patient's State So Severe?

One of the evolutionary strengths of *T. gondii* is its ability to adapt its reproductive behaviour in different environments. In North America and Western Europe, it benefits from ancient agricultural habits of breeding of a limited number of species, which provide a stable environment. The latter is ideal for asexual reproduction, thus leading to clonal propagation of a few successful lineages [5]. In these areas, 95% of *T. gondii* strains belong to three main lineages named type I, II, and III.

On the contrary, the Amazonian rainforest ecosystem is a hotbed of diversity. In order to survive in such an environment, *T. gondii* uses preferentially sexual reproduction in wild felids' gastrointestinal tract to generate atypical strains with highly divergent genotypes [5]. The report of an atypical *T. gondii* strain isolated in a free-living jaguar in French Guiana supports the existence of a *T. gondii* wildlife cycle and then genetic diversity [6]. Since 1998, several severe (and sometimes lethal) cases of acute toxoplasmosis in immunocompetent subjects involving such strains have been reported in French Guiana [7]–[10]. They disseminate via parasitaemia to multiple organs and especially to the lungs [10].

So far, there are only epidemiological and clinical data [10] supporting the fact that atypical Guianan strains are more virulent in immunocompetent patients than other *T. gondii* strains. Their high genetic diversity may provide them with enhanced invasive abilities, or it could interfere with the Th1 host immune response, as suggested by experiments in mouse models [11]–[13]. However, evidence has also been given indicating interindividual variability after infection to a same Guianan strain in an outbreak [14]. Biochemical mechanisms of virulence have not yet been studied in humans, but experimentally, ROP proteins seem to be essential for invasion and maintenance of the parasitophorous vacuole membrane. It has been shown that the overexpression of *ROP18* or the transfection of the *ROP18* allele into a nonpathogenic *T. gondii* strain enhances mortality in a mouse model [11]–[13]. Further studies, such as the *ROP18* gene sequencing of atypical Guianan strains, would be of high scientific value.

## Source of Infection

There is no formal evidence that the Maïpouri meat was the source of infection. Nonetheless, many strong arguments support this view. First, previous studies [9] demonstrated that consumption of game from the Amazonian forest was strongly associated with the risk of developing 10 to 20 days later severe toxoplasmosis. Second, we have proved that there was *T. gondii* DNA in the Maïpouri sample. Third, the patient ate undercooked meat, which makes highly likely the fact that he ingested living *T. gondii* cysts. Finally, and despite thorough questioning, we could not identify any other potential source of *T. gondii* infection. The patient was living in an urban area, without any pets, and has never travelled to the Amazonian rainforest. He drank only boiled water and did not report any other raw-meat consumption in the month before the beginning of symptoms besides the Maïpouri steak. To our knowledge, evidence has never been published demonstrating that a piece of meat was the source of human *Toxoplasma* infection by matching the parasite's genotype from the patient's biological sample and from a piece of meat likely to be the source of infection.

Among the five people who shared the Maïpouri dish, only two ate undercooked meat: the patient and his father. The latter remained asymptomatic, but he refused to perform a test to check his serological status against toxoplasmosis. Only hypotheses can be made to explain why he remained healthy. Either his steak was free of *T. gondii* cyst or most probably, thanks to his age, he was already immunized against toxoplasmosis.

## Conclusion

In medical practice, *T. gondii* is mainly a health concern in pregnant women and in immunosuppressed patients. In immunocompetent patients, acute toxoplasmosis is generally asymptomatic or associated with benign symptoms such as prolonged fever, polyadenopathy, and myalgia. Severe complications, such as pneumonia, myocarditis, or meningo-encephalitis, are infrequent. Eye involvement is likely underestimated, and the burden of ocular toxoplasmosis seems to be extremely high in certain tropical areas of South America [15].

To date, more than a hundred cases of acute toxoplasmosis in immunocompetent patients (adults and children) due to atypical strains of *T. gondii* have been reported in French Guiana (M. Demar, personal data). Approximately 40 have been published. It is highly likely that these atypical and virulent strains of *T. gondii* also exist in other Amazonian countries than French Guiana, but clinical cases are probably underreported.

Physicians should systematically consider acute toxoplasmosis as a possible diagnosis for any infectious syndrome with visceral (especially lung) involvement in patients who have recently travelled to or lived in the Amazonian area. Since lethal cases have been reported and since treatment associating pyrimethamine and sulfadiazine is efficient, such treatment should be promptly started immediately after serological results, without waiting for positive PCR or parasitic isolation.

**Key Learning Points**

- Atypical and highly virulent strains circulating in the Amazonian rainforest ecosystem
- To date only described in French Guiana but likely underreported in other countries of the Amazonian area
- Lung involvement is frequent and lethal cases have been described
- Specific anti-toxoplasmosis treatment should be promptly started immediately after serological results, without waiting for positive PCR or parasitic isolation
